# Effects of replacing soybean meal with enzymolysis-fermentation compound protein feed on growth performance, apparent digestibility of nutrients, carcass traits, and meat quality in growing-finishing pigs

**DOI:** 10.1186/s40104-024-01080-x

**Published:** 2024-09-12

**Authors:** Yu Cheng, Jun He, Ping Zheng, Jie Yu, Junning Pu, Zhiqing Huang, Xiangbing Mao, Yuheng Luo, Junqiu Luo, Hui Yan, Aimin Wu, Bing Yu, Daiwen Chen

**Affiliations:** Key Laboratory of Animal Disease-Resistant Nutrition, Ministry of Education, Animal Nutrition Institute, Sichuan Agricultural University, Ya’an, 625014 People’s Republic of China

**Keywords:** Compound protein feed, Enzymolysis-fermentation, Growing-finishing pigs, Growth performance, Nutrient digestibility

## Abstract

**Background:**

Addressing the shortage of high-quality protein resources, this study was conducted to investigate the effects of replacing soybean meal (SBM) with different levels of enzymolysis-fermentation compound protein feed (EFCP) in the diets of growing-finishing pigs, focusing on growth performance, nutrients digestibility, carcass traits, and meat quality.

**Methods:**

Sixty DLY (Duroc × Landrace × Yorkshire) pigs with an initial body weight of 42.76 ± 2.05 kg were assigned to 5 dietary treatments in a 2 × 2 + 1 factorial design. These dietary treatments included a corn-soybean meal diet (CON), untreated compound protein feed (UCP) substitution 50% (U50) and 100% SBM (U100) diets, and EFCP substitution 50% (EF50) and 100% SBM (EF100) diets. Each treatment had 6 pens (replicates) with 2 pigs per pen, and the experiment lasted 58 d, divided into phase I (1–28 d) and phase II (29–58 d). Following phase I, only the CON, U50, and EF50 groups were continued for phase II, each with 5 replicate pens. On d 59, a total of 15 pigs (1 pig/pen, 5 pens/treatment) were euthanized.

**Results:**

During phase I, the EF50 group had a higher average daily gain (ADG) in pigs (*P* < 0.05) compared to the CON group, whereas the U50 group did not have a significant difference. As the substitution ratio of UCP and EFCP increased in phase I, there was a noticeable reduction in the final body weight and ADG (*P* < 0.05), along with an increase in the feed-to-gain ratio (F/G) (*P* < 0.05). In phase II, there were no significant differences in growth performance among the treatment groups, but EF50 increased the apparent digestibility of several nutrients (including dry matter, crude protein, crude fiber, acid detergent fiber, ash, gross energy) compared to U50. The EF50 group also exhibited significantly higher serum levels of neuropeptide Y and ghrelin compared to the CON and U50 groups (*P* < 0.05). Moreover, the EF50 group had higher carcass weight and carcass length than those in the CON and U50 groups (*P* < 0.05), with no significant difference in meat quality.

**Conclusions:**

The study findings suggest that replacing 50% SBM with EFCP during the growing-finishing period can improve the growth performance, nutrient digestibility, and carcass traits of pigs without compromising meat quality. This research offers valuable insights into the modification of unconventional plant protein meals and developing alternatives to SBM.

## Background

Soybean meal (SBM) is commonly used in the global feed industry due to its high protein content and well-balanced amino acid (AA) composition [1, 2]. However, the rising prices of SBM and escalating competition for feed ingredients between humans and monogastric animals pose significant challenges to the livestock industry’s economic viability and long-term growth. To address these challenges, it is crucial to explore alternative plant proteins as substitutes for SBM.

Unconventional plant protein meals (UPPMs), such as rapeseed meal (RSM), cottonseed meal (CSM), and brewer’s spent grains (BSG), are used in animal feed due to their cost-effectiveness and wide availability [3, 4]. RSM is rich in protein, and sulfur-containing AA, and the AA profile is well-balanced [5, 6]. However, the presence of glucosinolates (GLs), phytic acid, fiber, and other anti-nutritional factors (ANFs) [5, 7, 8] limits its inclusion in animal diets, due to potential toxicity risks, reduced nutrient digestibility, and impaired growth performance [9–14]. CSM, a by-product of cottonseed oil production, contains a crude protein (CP) content ranging from 30% to 50%, and is rich in various AA, vitamin B, mineral elements, and carbohydrates [15, 16], making it an economically viable substitute for SBM. Nevertheless, its application in monogastric animals is restricted by ANFs such as free gossypol (FG), phytin, cyclopropene fatty acids, crude fiber (CF), and others [17], which can negatively impact growth performance, feed conversion, and fertility, and also cause abnormalities in intestinal development and internal organs [18–22]. BSG, a by-product of beer manufacturing, contains approximately 70% fiber, 20% CP, 10% lipids, as well as AAs, vitamins, minerals, and phenolic compounds [23–25]. Despite being used in cattle [26], poultry [27, 28], pig [29], and fish feed [30], its degradation rate remains limited due to its high fiber content (including 28.35% hemicellulose, 16.25% cellulose, and 7.27% lignin) [31], thus restricting its broader application. To overcome these limitations and enhance the applicability of CSM, RSM, and BSG in animal feed, pretreatment of these UPPMs is essential.

Microbial fermentation and enzymolysis are two primary methods used to reduce the ANFs (e.g., GLs, FG, and fibers) and improve the nutritional value of UPPMs [32–36]. These approaches are known for their environmental friendliness, energy efficiency, and cost-effectiveness. Microbial fermentation, which typically involving the use of fungi, yeast, and bacteria, can degrade ANFs and macromolecules (proteins, fibers) through the action of enzymes released by rapidly growing microorganisms [4, 37]. However, the hydrolysis rate during fermentation is very slow [38], leading to a time-consuming process (often lasting 48 h or more) [39, 40], possibly due to inadequate enzyme secretion by microorganisms during fermentation. Enzymolysis entails directly adding commercial enzymes to specificity degrade macromolecules and ANFs. Nevertheless, the enzymes currently utilized, mainly non-starch polysaccharide enzymes and protease [41–43] are not sufficiently efficient, possibly due to the presence of ANFs [39]. From the highlights and challenges of microbial fermentation and enzymolysis methods discussed above, it is evident that while each pretreatment method makes a significant contribution individually, no single method yields efficient results with its inherent limitations. Therefore, the combination of both pretreatment strategies could mitigate these drawbacks effectively, ultimately resulting in the desired outcomes. Combining enzymolysis with microbial fermentation has been demonstrated to better improve the nutritional quality of UPPMs. For example, Li et al. [34] found that pretreating RSM with protease enzymolysis and *Bacillus subtilis* fermentation resulted in more significant effects on increased peptides and organic acids content, while decreasing GLs and erucic acid content, compared to RSM treated with only enzymolysis or only fermentation.

At present, the research mainly focuses on the modification and application of individual UPPM, which have a less balanced AA composition compared to SBM. To improve the utilization rate of UPPMs, there are mainly two different methods that can be used. One is to directly add crystalline AAs, and the other alternative method is to compound UPPMs based on their individual AA content and proportion, to alleviate their nutritional deficiencies. In this study, we formulated RSM-CSM-BSG compound protein feeds by combining RSM (with low arginine content), CSM (with high arginine content), and BSG (with high nitrogen-free extract content) in a ratio of 45%:40%:15%. Additionally, there is limited literature on the effect of mixed UPPMs feed pretreated with enzymes and probiotics in vitro; as well as few studies on the application of enzymolysis-fermentation UPPMs in growing-finishing pigs.

It’s known that fermented or enzymolysis feeds can improve animal growth performance [40, 44], nutrient digestibility [40], carcass traits [45], and meat quality [44, 46]. Based on previous studies, it’s hypothesized that the combination of enzymolysis and fermentation can improve the quality and feeding efficiency of UPPMs. Therefore, this study was conducted to investigate the effects of combining complex enzymes and *Lactobacillus plantarum* on the nutritional values and ANFs of RSM-CSM-BSG compound protein feeds. The study also aimed to assess the potential for replacing SBM with enzymolysis-fermentation RSM-CSM-BSG compound protein feeds in growing-finishing pig diets. The objective was to establish a theoretical foundation for the broader application of UPPMs.

## Materials and methods

### Preparation of enzymolysis-fermentation compound protein feed

*Lactobacillus plantarum* strain was sourced from Beijing Beina Chuanglian Biotechnology Institute (Beijing, China). After the strain activation, a single colony was inoculated into 600 mL de Man, Rogosa, Sharpe broth (Hope Biotechnology Co., Ltd., Qingdao, China) in a 1000-mL Erlenmeyer flask and cultured statically at 37 °C for 24 h. Subsequently, the absorbance value (OD_600_) of the solution was adjusted to 1.4–1.5 for future use. Enzymes including cellulase (1 × 10^4^ U/g), xylanase (2 × 10^5^ U/g), pectinase (3 × 10^4^ U/g), and β-glucanase (3 × 10^4^ U/g) were purchased from Bestzyme Bio-Engineering Co., Ltd. (Shandong, China). Alkaline protease, neutral protease, and acid protease with enzyme activities of 2 × 10^6^ U/g, 5 × 10^5^ U/g, and 5 × 10^5^ U/g respectively, were obtained from Qingdao GBW Group Co., Ltd. (Shandong, China).

The compound protein feed (CPF) consisted of 45% RSM, 40% CSM, and 15% BSG. For the preparation of enzymolysis-fermentation compound protein feed (EFCP), the CPF was hydrolyzed with 0.6% of complex enzymes (including 12 U/g cellulase; 120 U/g xylanase; 9 U/g pectinase; 3 U/g β-glucanase; 300 U/g alkaline protease; 37.5 U/g neutral protease; 37.5 U/g acidic protease) for 8 h under a feed to water ratio of 1:2 at a temperature 55 °C. Then, each kilogram of CPF was inoculated 60 mL *Lactobacillus plantarum* and fermented at 37–40 °C for 16 h.

### Experimental design and diets

A total of 60 growing-finishing pigs (Duroc × Landrace × Yorkshire, DLY) with an initial body weight of 42.76 ± 2.05 kg were used in a 2 × 2 + 1 factorial experiment. The two factors were: the proportion of SBM replaced by CPF (50% vs. 100%) and the source of the CPF (untreated compound protein feed (UCP) vs. EFCP). Pigs were allocated using a randomized complete block design, with the initial body weight as the blocking factor. Within blocks, pigs were assigned to 5 dietary treatments consisting of a corn-soybean meal basal diet (CON), UCP substitution 50% (U50) and 100% SBM (U100) diets, and EFCP substitution 50% (EF50) and 100% SBM (EF100) diets. Each treatment consisted of 6 pens (replicates) with 2 pigs per pen. The feeding experiment lasted 58 days which were divided into phase I (1–28 d) and phase II (29–58 d). After phase I (1–28 d), only three treatment groups CON, U50, and EF50 were continued for phase II (29–58 d), each with 5 replicate pens. The diets for phase I and phase II were formulated according to the NRC (2012) [47], as detailed in Table 1.

**Table 1:** Composition and nutrient levels of experimental diets (air dry basis, %)

Ingredient	Phase I: 40–75 kg					Phase II: 75–100 kg		
	CON^a^	U50	U100	EF50	EF100	CON	U50	EF50
Corn	74.23	74.23	74.23	74.23	74.23	85.31	85.46	85.46
Soybean meal	16.77	8.39	0.00	8.39	0.00	9.20	4.60	4.60
Wheat bran	2.00	1.05	0.00	1.05	0.00	1.00	0.30	0.30
Unite Bran	1.69	0.82	0.00	0.82	0.00	1.00	0.55	0.55
UCP^b^	0.00	10.15	20.29	0.00	0.00	0.00	5.57	0.00
EFCP^c^	0.00	0.00	0.00	10.15	20.29	0.00	0.00	5.57
Soybean oil	1.80	1.80	1.80	1.80	1.80	0.70	0.70	0.70
Limestone	0.60	0.57	0.54	0.57	0.54	0.44	0.43	0.43
Dicalcium phosphate	1.27	1.22	1.20	1.22	1.20	1.00	0.95	0.95
NaCl	0.30	0.30	0.30	0.30	0.30	0.30	0.30	0.30
L-Lysine·HCl	0.60	0.72	0.84	0.72	0.84	0.46	0.53	0.53
DL-Methionine	0.10	0.09	0.09	0.09	0.09	0.03	0.03	0.03
L-Tryptophane	0.06	0.07	0.08	0.07	0.08	0.04	0.05	0.05
L-Threonine	0.20	0.22	0.25	0.22	0.25	0.14	0.15	0.15
50% Choline chlorine	0.15	0.15	0.15	0.15	0.15	0.15	0.15	0.15
Vitamin premix^d^	0.03	0.03	0.03	0.03	0.03	0.03	0.03	0.03
Mineral premix^e^	0.20	0.20	0.20	0.20	0.20	0.20	0.20	0.20
Total	100	100	100	100	100	100	100	100
Nutrient levels^f^								
DE, MJ/kg	14.02	13.81	13.55	13.81	13.55	13.97	13.84	13.84
CP	14.24	14.24	14.24	14.24	14.24	11.50	11.50	11.50
Ca	0.63	0.63	0.63	0.63	0.63	0.49	0.49	0.49
AP	0.29	0.29	0.29	0.29	0.29	0.23	0.23	0.23
D-Lys	1.01	1.01	1.01	1.01	1.01	0.74	0.74	0.74
D-Met	0.30	0.30	0.30	0.30	0.30	0.21	0.21	0.21
D-Thr	0.61	0.61	0.61	0.61	0.61	0.47	0.47	0.47
D-Trp	0.18	0.18	0.18	0.18	0.18	0.13	0.13	0.13

^a﻿^CON: Control diet based on corn and soybean meal, U50 and U100 diets were made by UCP substituting for 50% and 100% soybean meal, EF50 and EF100 diets were made by EFCP substituting for 50% and 100% soybean meal

^b^UCP : Untreated compound protein feed

^c^EFCP: Enzymolysis-fermentation compound protein feed

^d^Vitamin premix provided the following per kg of diets: vitamin A, 9,000 IU; Vitamin D_3_, 3,000 IU; Vitamin E, 24 IU; Vitamin K_3_, 3 mg; Vitamin B_1_, 3 mg; Vitamin B_2_, 7.5 mg; Vitamin B_6_, 3.6 mg; Vitamin B_12_, 0.36 mg; D-Biotin, 1.5 mg; D-Pantothenic acid, 15 mg; Folic acid, 1.5 mg; Nicotinamide, 30 mg

^e^Mineral premix provided the following per kg of diets: Fe (FeSO_4_·H_2_O), 50 mg; Cu (CuSO_4_·5H_2_O), 10 mg; Mn (MnSO_4_·H_2_O), 4 mg; Zn (ZnSO_4_·H_2_O), 50 mg; I (KI), 0.3 mg; Se (Na_2_SeO_3_), 0.3 mg

^f^Dietary nutrient levels were calculated values

### Feeding management

Pigs were housed in a controlled environment room with 36 pens (2.0 m × 3.0 m). The room was equipped with a temperature-controlled system, to maintain temperatures ranging from 22 to 28 °C for each phase. The diets provided to pigs were liquid diets, which were prepared immediately before feeding by mixing dry feed and water in a 1:2 ratio. During the experimental period, pigs were fed two times daily, at 8:00 and 16:00. All pigs were individually weighed at the 28 and 59^th^ days of the experiment after 12 h of fasting, to calculate the average daily gain (ADG). Feed intake was measured by pen, and the average daily feed intake (ADFI) was calculated by dividing the total feed intake of each pen by the number of pig-days in that pen. The feed-to-gain ratio (F/G) was then calculated by dividing ADFI by ADG.

### Sample collection

During phase II, fecal samples were collected in self-sealing bags for 4 consecutive days (from d 44 to 47). For every 100 g of fresh feces, 10 mL of 10% dilute sulfuric acid and two drops of toluene were added, thoroughly mixed, and stored at −20 °C. At the end of the experiment, the feces were mixed according to treatment and oven-dried at 65 °C to a constant weight, then smashed to pass through a 1.0-mm screen for chemical analysis. In the morning of d 59 after fasting for 12 h, blood samples were obtained via *anterior vena cava* puncture and collected into the non-anticoagulative tube. Serum was collected after centrifugation at 3,500 r/min for 15 min at 4 °C and stored at −20 °C until analysis. After blood collection, 15 pigs (1 pig/pen, 5 pens/treatment) were slaughtered in an industrial slaughterhouse. Samples of the longissimus dorsi muscle (LDM) were used for meat quality and intramuscular fat (IMF) content analysis.

### Analysis of carcass traits

Following slaughter, the hot carcass weight of each pig was measured and used to calculate the dressing percentage. The carcass length was defined as the distance between the united phalanges and the first cervical vertebra. The backfat thickness at the thickest part of the shoulder, thoracolumbar junction, and lumbar-sacral junction were recorded and used to calculate the average backfat value. The fat thickness and muscle thickness at the penultimate 3–4 ribs were recorded and a formula was used to calculate lean meat rate. Loin muscle area was measured at the tenth rib on the right side of carcass.

### Measurement of meat quality

The evaluation of meat quality was conducted following established protocols [48]. Briefly, meat color (brightness, L*; redness, a*; yellowness, b*) was measured 45 min and 24 h after slaughter using a colorimeter (NR10QC, 3nh, Shenzhen, China). The pH values of the meat at 45 min and 24 h post-slaughter were determined using a calibrated pH meter (testo 205, Testo Inc, Lenzkirch, Germany). Cooking loss was calculated by measuring the weight change of muscle samples before and after cooking, starting from 45 min post-slaughter. The method for determining the drip loss percentage followed previous descriptions [49]. Approximately 45 min postmortem, a cuboid (5 cm × 3 cm × 2.5 cm) weighing about 30 g was manually trimmed from the LDM and weighed. This sample was then suspended in an inflated plastic bag at 2–4 °C and weighed after 24 h. Drip loss was quantified as the percentage of weight change. The IMF content of samples was determined by Soxhlet extraction.

### Chemical analysis

The contents of dry matter (DM), CP, ether extract (EE), CF, neutral detergent fiber (NDF), ash, and acid insoluble ash (AIA) in UCP, EFCP, fecal and diets were analyzed according to the national standards of the People’s Republic of China GB/T 6435–2014 [50], GB/T 6432–2018 [51], GB/T 6433–2006 [52], GB/T 6434–2006 [53], GB/T 20806–2006 [54], GB/T 6438–2007 [55] and GB/T 23742–2009 [56], respectively. The acid detergent fiber (ADF) was determined following the agricultural industry standard of the People’s Republic of China, NY/T1459–2022 [57]. The gross energy (GE) of all samples was determined using an adiabatic oxygen bomb calorimeter (Parr6400 Instrument Co., Moline, IL, USA). The trichloroacetic acid-soluble protein (TCA-SP) was measured according to the agricultural industry standard of the People’s Republic of China, NY/T 3801–2020 [58]. The peptides were calculated by subtracting free amino acids from TCA-SP. The AA profiles of UCP and EFCP were analyzed using an AA analyzer (L-8800; Hitachi, Tokyo, Japan). The content of GLs was determined following the agricultural industry standard of the People’s Republic of China, NY/T 1582–2007 [59]. The content of FG, isothiocyanates (ITC), and oxazolidinethione (OZT) were ascertained referencing the national standards of the People’s Republic of China GB/T 13086–2020 [60], GB/T 13087–2020 [61], and GB/T 13089–2020 [62], respectively. AIA was used as an endogenous indicator and the endogenous indicator method was used to calculate the apparent nutrient digestibility.

### Calculations

The calculation formulas for apparent nutrient digestibility and dressing precentage are as follows:

$$\begin{aligned}&\mathrm{Apparent}\;\mathrm{nutrient}\;\mathrm{digestibility}\;\left(\%\right)=100-\left[\left(\mathrm{AIA}\;\mathrm{content}\;\mathrm{in}\;\mathrm{the}\;\mathrm{feed}/\mathrm{AIA}\;\mathrm{content}\;\mathrm{in}\;\mathrm{the}\;\mathrm{fecal}\right) \right. \\ & \left. \times\left(\mathrm{the}\;\mathrm{content}\;\mathrm{of}\;\mathrm a\;\mathrm{nutrient}\;\mathrm{in}\;\mathrm{the}\;\mathrm{fecal}/\mathrm{the}\;\mathrm{content}\;\mathrm{of}\;\mathrm a\;\mathrm{nutrient}\;\mathrm{in}\;\mathrm{the}\;\mathrm{feed}\right)\right]\times100.\\ \\ & \mathrm{Dressing}\;\mathrm{percentage}\;\left(\%\right)=\left(\mathrm{carcass}\;\mathrm{weight}/\mathrm{live}\;\mathrm{weight}\;\mathrm{at}\;\mathrm{slaughter}\right)\times100. \end{aligned}$$

### Serum parameters

The concentrations of alanine aminotransferase (ALT), aspartate aminotransferase (AST), total protein (TP), albumin (ALB), alkaline phosphatase (ALP), glucose (GLU), urea (UREA), triglyceride (TG), total cholesterol (TC), low-density lipoprotein cholesterol (LDL-C) and high-density lipoprotein cholesterol (HDL-C) in serum were determined using the fully automatic biochemistry analyzer 3100 (Hitachi, Japan) and the kits used in the analyzer were obtained from Maccura Biotechnology Co., Ltd. (Chengdu, China).

The levels of serum immunoglobulin A (IgA), triiodothyronine (T3), thyroxine (T4), leptin, cholecystokinin (CCK), ghrelin, neuropeptide Y (NPY), tumor necrosis factor-α (TNF-α), interleukin-6 (IL-6), interleukin-1β (IL-1β), interferon-γ (IFN-γ), interleukin-4 (IL-4), and interleukin-10 (IL-10) were determined using the corresponding ELISA kits (Jiangsu Meimian Industrial Co., Ltd., Jiangsu, China). The assay was based on the double-antibody sandwich method, and all procedures were conducted following the provided instruction manual.

### Statistical analysis

Data were analyzed using SAS 9.4 (SAS Institute, Inc., Cary, NC, USA). For phase I, the experimental data were subjected to both one-way ANOVA and two-way ANOVA using the Mixed model. The primary factors in the model included the proportion of CPF replacement and the source of the CPF, as well as their interaction. For phase II, data were exclusively analyzed by one-way ANOVA. Multiple comparisons were performed using the LSD method. Results are presented as means with their corresponding standard error of the mean (SEM). A value of *P* < 0.05 was considered statistically significant, while 0.05 ≤ *P* ≤ 0.10 was regarded as a significant trend.

## Results

### Nutrient composition of UCP and EFCP

The nutrient composition of UCP and EFCP were shown in Tables 2, 3, and 4. In EFCP, the contents of TCA-SP, peptides, and total free amino acids were higher by 261.75%, 300.00%, and 164.24%, respectively, compared to those in UCP. Conversely, the total amino acid in EFCP (34.79%) was lower than that in UCP (35.65%). Concentrations of CF, NDF, ADF, and GLs in EFCP decreased by 10.52%, 45.96%, 6.52%, and 94.92%, respectively, compared with UCP. ITC was not detected. However, the content of FG in the EFCP was elevated by 210.34% more than that in the UCP.

**Table 2:** Nutrient and anti-nutritional factor contents of CPF before and after enzymolysis-fermentation (dry matter basis)

Items	UCP	EFCP
CP, %	41.52	42.27
TCA-SP, %	5.36	19.39
Peptides, %	3.85	15.40
EE, %	2.75	2.63
CF, %	17.30	15.48
NDF, %	53.85	29.10
ADF, %	29.43	27.51
Ash, %	8.37	8.69
GLs, μmol/g	21.07	1.07
ITC, mg/kg	513.45	ND
OZT, mg/kg	ND	ND
FG, mg/kg	134.89	418.62

*UCP* Untreated compound protein feed, *EFCP* Enzymolysis-fermentation compound protein feed, *CP* Crude protein, *TCA-SP* Trichloroacetic acid-soluble protein, *EE* Ether extract, *CF* Crude fiber, *NDF* Neutral detergent fiber, *ADF* Acid detergent fiber, *GLs* Glucosinolates, *ITC* Isothiocyanates, *OZT* Oxazolidinethione, *FG* Free gossypol, *ND* Not detected

**Table 3:** Amino acid content of CPF before and after enzymolysis-fermentation (dry matter basis, %)

Items	UCP	EFCP
Indispensable AA		
Arg	3.21	2.50
His	1.27	1.17
Ile	1.46	1.47
Leu	2.44	2.52
Lys	1.86	1.65
Met	0.35	0.40
Phe	2.07	2.05
Thr	1.40	1.36
Trp	0.40	0.37
Val	1.97	2.03
Dispensable AA		
Gly	1.78	1.81
Ser	1.38	1.27
Ala	2.16	2.23
Asp	3.12	3.17
Cys	0.34	0.24
Glu	7.48	7.61
Pro	1.89	1.91
Tyr	1.07	1.03
Total amino acids	35.65	34.79

*UCP* Untreated conpound protein feed, *EFCP* Enzymolysis-fermentation compound protein feed

**Table 4:** Free amino acid content of CPF before and after enzymolysis-fermentation (dry matter basis, %)

Items	UCP	EFCP
Indispensable AA		
Arg	0.27	0.06
His	0.02	0.07
Ile	0.03	0.07
Leu	0.02	0.39
Lys	0.02	0.09
Met	0.00	0.11
Phe	0.03	0.45
Thr	0.06	0.20
Val	0.11	0.23
Dispensable AA		
Gly	0.03	0.12
Ser	0.02	0.18
Ala	0.56	0.77
Asp	0.16	0.24
Cys	0.02	0.12
Glu	0.11	0.51
Pro	0.03	0.05
Tyr	0.02	0.33
Total free amino acids	1.51	3.99

*UCP* Untreated compound protein feed, *EFCP* Enzymolysis-fermentation compound protein feed

### Growth performance

The effects of replacing SBM with EFCP on the growth performance are shown in Table 5. During phase I, it was observed that the source of the CPF (EFCP vs. UCP) had no significant effect on growth performance. However, as the replacement ratio of SBM with EFCP increased, there was a significant decrease (*P* < 0.05) in ADG and body weight on d 28, accompanied by an increase (*P* < 0.05) in the F/G. There was no interaction between the substitution ratio of CPF and the source of CPF on growth performance. The EF50 group exhibited a higher ADG compared to the CON group (*P* < 0.05). Both U100 and EF100 groups showed no significant difference in body weight on d 28, ADG, and ADFI when compared to the CON group; however, their F/G were significantly higher (*P* < 0.05).

**Table 5:** Effects of replacing soybean meal with EFCP on the growth performance of growing-finishing pigs (1–58 d)

Items	Dietary treatment^1^					SEM	*P*_1_^2^	*P*_2_^3^		
	CON	U50	U100	EF50	EF100			EF	SUB	EF × SUB
D 1 to 28 (*n* = 6)										
Initial BW, kg	42.08	42.42	42.63	43.33	43.33	0.89	0.81	0.36	0.91	0.91
BW on d 28, kg	74.88	76.75	73.79	79.04	75.63	1.56	0.19	0.16	0.04	0.87
ADG, kg/d	1.17^bc^	1.23^ab^	1.11^c^	1.28^a^	1.16^bc^	0.04	0.04	0.23	< 0.01	0.95
ADFI, kg/d	2.48	2.61	2.53	2.67	2.57	0.08	0.58	0.55	0.27	0.93
F/G	2.12^c^	2.13^bc^	2.27^a^	2.09^c^	2.22^ab^	0.04	< 0.01	0.29	< 0.01	0.95
D 29 to 58 (*n *= 5)										
BW on d 29, kg	76.10	77.40		80.55		1.38	0.10			
Final BW, kg	103.40	103.15		109.25		2.42	0.17			
ADG, kg/d	0.91	0.86		0.96		0.06	0.54			
ADFI, kg/d	3.01	3.01		3.18		0.11	0.49			
F/G	3.34	3.52		3.38		0.14	0.66			

Values are expressed as the mean of all replicates in each treatment group (d 1 to 28, *n* = 6; d 29 to 58, *n* = 5)

^1^CON: Control diet based on corn and soybean meal; U50 and U100 diets were made by UCP substituting for 50% and 100% soybean meal; EF50 and EF100 diets were made by EFCP substituting for 50% and 100% soybean meal

^2^*P*_1_ represents the *P* value of one-way ANOVA among five or three different groups

^3^*P*_2_ indicated the two-way ANOVA *P* value of compound protein feed source and compound protein feed substitution ratio. EF: Compound protein feed source effect; SUB: Compound protein feed substitution ratio effect; EF × SUB: Interaction effect of compound protein feed source and compound protein feed substitution ratio

^a–c^Different lowercase letters indicate significant differences between groups (LSD test after one-way ANOVA, *P*<0.05)

In phase II, there were no significant differences in growth performance among the treatment groups. However, the final body weight of the EF50 group was numerically higher than that of the CON and U50 groups.

### Apparent digestibility of nutrients

The effects of replacing SBM with EFCP on the apparent digestibility of nutrients are presented in Table 6. The U50 group showed lower digestibility of DM, CP, CF, NDF, ADF, ash, and GE compared to the CON group (*P* < 0.05). Additionally, except for EE and NDF, the apparent digestibility of other nutrients in the U50 group was significantly lower (*P* < 0.05) than that in the EF50 group. In the EF50 group, the digestibility of EE, CF, and ash was significantly higher (*P* < 0.05), while that of NDF was significantly lower (*P* < 0.05) compared to the CON group. However, no significant differences were observed in the digestibility of other nutrients between the EF50 and CON groups.

**Table 6:** Effects of replacing soybean meal with EFCP on apparent nutrient digestibility of growing-finishing pigs in the phase II

Items, %	Dietary treatment^1^			SEM	*P*-value
	CON	U50	EF50		
DM	88.67^a^	84.96^b^	88.79^a^	0.35	< 0.01
CP	81.65^a^	75.40^b^	81.98^a^	0.93	< 0.01
EE	80.32^b^	81.78^ab^	83.20^a^	0.52	< 0.01
CF	40.59^b^	26.23^c^	57.90^a^	2.99	< 0.01
NDF	82.42^a^	78.73^b^	79.82^b^	0.70	< 0.01
ADF	58.72^a^	43.92^b^	59.59^a^	2.90	< 0.01
Ash	47.71^b^	31.22^c^	53.72^a^	1.40	< 0.01
GE	87.91^a^	84.04^b^	88.57^a^	0.38	< 0.01

Values are expressed as the mean of all replicates in each treatment group (*n* = 5)

^1^CON: Control diet based on corn and soybean meal; U50 diets were made by UCP substituting for 50% soybean meal; EF50 diets were made by EFCP substituting for 50% soybean meal

^a−c^The shoulder label without letters or the same lowercase letters indicated that the difference was not significant (*P* ≥ 0.05), and different lowercase letters indicated significant differences (*P* < 0.05)

### Carcass traits

As presented in Table 7, the U50 group had no significant effects on carcass traits compared with the CON group. However, the EF50 group had significantly higher carcass weight and length (*P* < 0.05) than those in the CON and U50 groups.

**Table 7:** Effects of replacing soybean meal with EFCP on carcass traits of growing-finishing pigs

Items	Dietary treatment^1^			SEM	*P*-value
	CON	U50	EF50		
Slaughter weight, kg	104.00^b^	102.50^b^	109.40^a^	1.31	< 0.01
Carcass weight, kg	69.64^b^	68.72^b^	73.16^a^	0.82	< 0.01
Carcass length, cm	81.08^b^	80.60^b^	83.30^a^	0.70	0.04
Dressing percentage, %	71.92	72.04	71.94	0.58	0.99
Average backfat thickness, cm	1.77	1.81	1.91	0.10	0.61
Lean meat rate, %	55.20	55.77	56.12	1.21	0.86
Loin muscle area, cm^2^	46.84	44.33	47.95	2.40	0.57

Values are expressed as the mean of all replicates in each treatment group (*n* = 5)

^1^CON: Control diet based on corn and soybean meal; U50 diets were made by UCP substituting for 50% soybean meal; EF50 diets were made by EFCP substituting for 50% soybean meal

^a–b^The shoulder label without letters or the same lowercase letters indicated that the difference was not significant (*P *≥ 0.05), and different lowercase letters indicated significant differences (*P *< 0.05)

### Meat quality

The result of meat quality is shown in Table 8, the b*_45min_ value was significantly lower (*P* < 0.05) in the U50 and EF50 groups compared to the CON group. However, there were no significant differences observed in the other indexes among the treatment groups.

**Table 8:** Effects of replacing soybean meal with EFCP on meat quality of growing-finishing pigs

Items	Dietary treatment^1^			SEM	*P*-value
	CON	U50	EF50		
pH_45min_	6.52	6.16	6.21	0.12	0.10
pH_24h_	5.52	5.48	5.54	0.03	0.33
L*_45min_	49.77	47.61	46.19	1.60	0.32
a*_45min_	5.82	5.09	5.67	0.48	0.55
b*_45min_	11.64^a^	10.39^b^	10.25^b^	0.28	< 0.01
L*_24h_	59.85	62.74	58.53	1.25	0.09
a*_24h_	8.82	8.85	9.97	0.65	0.39
b*_24h_	13.61	14.09	13.06	0.37	0.19
Cooking loss, %	31.11	32.70	32.93	1.02	0.42
Drip loss, %	1.97	2.37	3.03	0.34	0.12
Intramuscular fat, %	3.51	3.51	2.93	0.25	0.39

Values are expressed as the mean of all replicates in each treatment group (*n* = 5)

^1^CON: Control diet based on corn and soybean meal; U50 diets were made by UCP substituting for 50% soybean meal; EF50 diets were made by EFCP substituting for 50% soybean meal

^a,b^The shoulder label without letters or the same lowercase letters indicated that the difference was not significant (*P* ≥ 0.05), and different lowercase letters indicated significant differences (*P* < 0.05)

### Serum biochemistry

According to Table 9, there were no significant differences in serum biochemical parameters among treatment groups. The U50 group showed no significant differences in serum levels of inflammatory factors compared to the CON group. However, the level of IL-6 was significantly higher in the EF50 group than in the CON group (*P* < 0.05).

**Table 9:** Effects of replacing soybean meal with EFCP on serum biochemical parameter on d 59 of growing-finishing pigs

Items	Dietary treatment^1^			SEM	*P-*value
	CON	U50	EF50		
ALT, U/L	42.93	46.82	42.67	5.44	0.84
AST, U/L	27.56	26.68	24.26	1.74	0.72
TP, g/L	60.70	58.12	58.65	1.17	0.29
ALB, g/L	30.49	29.98	30.93	0.77	0.69
ALP, U/L	97.80	95.60	102.40	9.74	0.88
GLU, mmol/L	5.26	4.73	4.66	0.33	0.40
UREA, mmol/L	1.70	1.95	1.95	0.17	0.51
LDL-C, mmol/L	1.05	1.13	1.27	0.12	0.42
HDL-C, mmol/L	0.72	0.70	0.70	0.06	0.97
TG, mmol/L	0.35	0.49	0.43	0.07	0.39
IgA, μg/mL	33.22	37.30	40.83	2.30	0.10
T3, pmol/L	201.84	214.99	232.08	12.83	0.29
T4, pmol/L	1,362.42	1,263.02	1,406.00	116.96	0.68
TNF-α, pg/mL	134.00	121.76	139.62	6.82	0.21
IL-6, pg/mL	502.33^b^	536.17^ab^	625.33^a^	31.87	< 0.05
IL-1β, pg/mL	460.81	476.89	512.84	24.70	0.35
IFN-γ, pg/mL	30.80	29.17	27.61	1.81	0.48
IL-4, pg/mL	41.36	43.71	49.39	3.61	0.31
IL-10, pg/mL	107.92	97.92	109.88	3.92	0.11

Values are expressed as the mean of all replicates in each treatment group (*n* = 5)

^1^CON: Control diet based on corn and soybean meal; U50 diets were made by UCP substituting for 50% soybean meal; EF50 diets were made by EFCP substituting for 50% soybean meal

^a,b^The shoulder label without letters or the same lowercase letters indicated that the difference was not significant (*P* ≥ 0.05), and different lowercase letters indicated significant differences (*P* < 0.05)

### Serum appetite-regulating hormones

The levels of appetite-regulating hormones in serum on d 59 are shown in Table 10. There were no significant differences in the levels of leptin, CCK, NPY, and ghrelin between the CON group and the U50 group. Additionally, the EF50 group demonstrated significantly higher levels of NPY and ghrelin compared to both CON and U50 groups (*P* < 0.05).

**Table 10:** Effects of replacing soybean meal with EFCP on the level of appetite-regulating hormones in serum on d 59 of growing-finishing pigs

Items	Dietary treatment^1^			SEM	*P*-value
	CON	U50	EF50		
Leptin, ng/L	1,487.50	1,683.16	2,033.85	194.09	0.17
CCK, ng/L	692.00	814.46	859.52	54.31	0.12
NPY, ng/L	269.53^b^	269.11^b^	393.80^a^	15.84	< 0.01
Ghrelin, ng/L	2,208.09^b^	2,413.99^b^	3,058.54^a^	201.10	0.03

Values are expressed as the mean of all replicates in each treatment group (*n* = 5)

^1^CON: Control diet based on corn and soybean meal; U50 diets were made by UCP substituting for 50% soybean meal; EF50 diets were made by EFCP substituting for 50% soybean meal

^a,b^The shoulder label without letters or the same lowercase letters indicated that the difference was not significant (*P* ≥ 0.05), and different lowercase letters indicated significant differences (*P* < 0.05)

## Discussion

SBM is a widely utilized protein ingredient in pig feed [63]. However, given the rising costs and fluctuating availability of SBM over the years, it urgently calls for the development of suitable alternatives to SBM. UPPMs are commonly used as a substitute for SBM, but their application in pig diets is limited by factors such as high fiber and lower protein contents, as well as the presence of ANFs (including CF, tannins, GLs, ITC, phytate, and FG) in UPPMs that can adversely affect feed digestibility and animal growth performance. Lee et al. [64] found that increasing dietary cold-pressed canola cake from 0 to 40% by reducing corn and SBM levels resulted in a linearly reduced FW, ADG, and ADFI, an increased F/G, and a reduction in the serum T4 level of pigs. Similarly, Velayudhan et al. [14] observed that increasing dietary expeller extracted canola meal from 0 to 30% led to a linear decrease in ADFI, an increase in thyroid weight and serum T3 level, while showing a linear reduction in serum T4, possibly due to GLs presented in expeller-extracted canola meal. Moreover, the replacement of soybean meal with UPPMs in pig diets can reduce nutrient digestibility [13, 65], and carcass traits [66], but usually has no adverse effect on meat quality [67, 68].

In order to enhance the utilization rate of UPPMs in pig diets and reduce the negative impacts, it is essential to employ processing technology to modify UPPMs. Currently, technologies such as microbial fermentation and enzymolysis are commonly employed for this purpose. Both techniques have been shown to increase CP content while simultaneously decreasing the content of CF, NDF, and ADF, as well as other ANFs presented in UPPMs [4, 34, 39, 69, 70]. In this experiment, a combined enzymolysis and microbial fermentation method was used to modify CPF. After adding non-starch polysaccharidases and proteases for enzymolysis over an 8-h period, followed by a 16-h fermentation with *Lactobacillus plantarum*, there was a significant increase in the content of TCA-SP, total free amino acids, and peptides in the EFCP. Meanwhile, the contents of CF, NDF, ADF, GLs, and ITC decreased. In general, microbial fermentation can reduce FG in the cottonseed meal [70, 71]. However, in our present study, it was interestingly found that the content of FG increased from 134.89 mg/kg to 418.62 mg/kg after enzymolysis-fermentation treatment. This phenomenon might be attributed to the addition of various proteases during the enzymolysis-fermentation process, which led to the degraded proteins in bound gossypol and the subsequent release of FG.

In the current study, replacing 50% SBM with EFCP during phase I was found to increase the ADG of pigs, which is in line with previous findings [40]. For instance, Tang et al. [72] found that diets fermented with *Lactobacillus plantarum, Pseudomonas prionis, Bacillus subtilis,* and *Aspergillus niger* significantly increased FW, ADG, and ADFI in pigs while concurrently decreased F/G. The observed increase in the ADG might be attributed to several factors. Firstly, the fermentation process of lactic acid bacteria would reduce the bitterness and astringency of the substrate and produce aromatic substances [73], thereby improving palatability of the EFCP, and promoting pig feed intake. Secondly, the enzymolysis-fermentation treatment degraded the complex proteins in EFCP into peptides or even AA, which can be more efficiently digested and absorbed by animals [74]. Moreover, the various ANFs in EFCP were extensively degraded by the enzymolysis-fermentation treatment. All of these factors contribute to the observed increase in ADG during phase I.

Intuerestingly, this study revealed that during phase I, replacing 50% of SBM with UCP did not have a negative impact on the growth performance of pigs compared to the CON group. However, in phase II, although UCP did not significantly affect growth performance, ADG was numerically reduced by 5.49% and F/G increased by 5.39% compared to the CON group. The observed effects may be attributed to the cumulative effects of various ANFs such as tannins, GLs, ITC, and FG present in the UCP with a longer feeding period. Additionally, replacing 50% of SBM with EFCP increased FW, ADG, and ADFI in phase II compared to the replacement with UCP. The increase in ADFI in pigs may be attributed to an increase in the serum level of appetite stimulators. In the current study, the levels of the appetite stimulators NPY and ghrelin in the EF50 group showed a significant increase, which may explain the elevation of ADFI in the EF50 group. Nakazato et al. [75] found that administering ghrelin to mice could promote feeding and increase body weight. Similarly, Gao et al. [76] observed that dietary supplementation of ghrelin could stimulate feed intake, growth, and *NPY* mRNA expression in grouper *Epinephelus coioides*. Given that ghrelin can enhance *NPY* gene expression [75, 76], and considering NPY which is a crucial factor for stimulating feed intake in mammals [77], may explain the observed increase in ADFI. Meanwhile, nutrient digestibility is an important factor influencing animal growth performance. The study found that improvement in growth performance corresponded with improved nutrient digestibility. These results are consistent with previous findings showing that the inclusion of fermented RSM or fermented CSM in pig diets can increase nutrient digestibility, resulting in improved pig growth performance [40, 74, 78].

Serum biochemical parameters serve as indicators of nutritional metabolism and the functional status of tissues and organs within an animal, providing valuable insights into the health status of pigs [79]. In this study, there were no significant differences in serum parameters and most inflammatory factors among the three diet groups. This finding is consistent with previous research, which reported that feeding fermented feeds improved the growth performance of pigs without affecting blood profile [80]. In short, replacing 50% of SBM with EFCP in the diets of growing-finishing pig did not lead to significant alterations in the serum biochemical parameters of pigs, demonstrating the feasibility and safety of using EFCP in pig diets to a certain extent in this study.

Carcass traits are important indicators of pig fattening efficiency. Previous studies have shown that supplementing pig diets with fermented feeds can improve carcass traits in finishing pigs [72, 81]. The results of this study revealed that replacing 50% of SBM with EFCP significantly increased carcass weight and length, which correlated with the highest FW in the EF50 group. Consumers heavily rely on meat color as a key indicator of freshness, wholesomeness, and quality at the point of sale, thereby influencing their purchase decisions [82, 83]. Therefore, meat color is a crucial determinant of meat quality and is typically assessed using L*, a*, and b* values. In this study, replacing 50% of SBM with either UCP or EFCP resulted in a reduction in the b*_45min_ value without affecting b*_24h_ or other meat color values. Generally, the b* value reflects the degree of browning in meat, which can make it less appealing [84]. The findings presented in this study suggested that EFCP can improve carcass traits without adversely impacting meat quality.

## Conclusion

Our findings indicated that enzymolysis-fermentation pretreatment of CPF resulted in an increase in the content of TCA-SP, free amino acids, and peptides, as well as a reduction in the content of CF, NDF, GLs, and ITC. Substituting 50% SBM with EFCP during the growing-finishing period improved growth performance, nutrient digestibility, and carcass traits without adverse effects on meat quality, and health status. These results could be used as a reference for developing high-quality protein feed resources to address challenges posed by the scarcity of high-quality protein resources. Furthermore, our study provided new perspectives and solutions for viable alternatives to SBM.

## Data Availability

The data used to support the findings of this study are available from the corresponding author upon request.

## Abbreviations

ANF: Anti-nutritional factor
AA: Amino acid
ADFI: Average daily feed intake
ADG: Average daily gain
ADF: Acid detergent fiber
AIA: Acid insoluble ash
AKP: Alkaline phosphatase
ALT: Alanine aminotransferase
AST: Aspartate aminotransferase
ALB: Albumin
BSG: Brewer’s spent grains
CON: Control diet based on corn and soybean mea
CP: Crude protein
CF: Crude fiber
CCK: Cholecystokinin
CSM: Cottonseed meal
CPF: Compound protein feed
DM: Dry matter
EFCP: Enzymolysis-fermentation compound protein feed
EF50: Diets were made by EFCP substituting for 50% soybean meal
EF100: Diets were made by EFCP substituting for 100% soybean meal
EE: Ether extract
F/G: Feed-to-gain ratio
FG: Free gossypol
GLs: Glucosinolates
GE: Gross energy
Glu: Glucose
HDL-C: High-density lipoprotein cholesterol
IMF: Intramuscular fat
ITC: Isothiocyanates
IL-1β: Interleukin1-β
IL-6: Interleukin-6
IFN-γ: Interferon-γ
IL-4: Interleukin-4
IL-10: Interleukin-10
IgA: Immunoglobulins A
LDM: Longissimus dorsi muscle
LDL-C: Low-density lipoprotein cholesterol
NPY: Neuropeptide Y
NDF: Neutral detergent fiber
OZT: Oxazolidinethione
RSM: Rapeseed meal
SBM: Soybean meal
SEM: Standard error of the mean
TCA-SP: Trichloroacetic acid-soluble protein
TP: Total protein
TG: Triglyceride
TC: Total cholesterol
T3: Triiodothyronine
T4: Thyroxine
TNF-α: Tumor necrosis factor-α
UPPM: Unconventional plant protein meals
UCP: Untreated compound protein feed
U50: Diets were made by UCP substituting for 50% soybean meal
U100: Diets were made by UCP substituting for 100% soybean meal
